# Advancements in Noninvasive Imaging: Optical Coherence Tomography for Evaluating Therapeutic Efficacy in Vitiligo

**DOI:** 10.1111/jocd.16771

**Published:** 2025-01-06

**Authors:** Isabella J. Tan, Sydney M. Wolfe, Bernard A. Cohen

**Affiliations:** ^1^ Robert Wood Johnson Medical School, Rutgers The State University of New Jersey Piscataway New Jersey USA; ^2^ Center for Dermatology Rutgers Robert Wood Johnson Medical School Somerset New Jersey USA; ^3^ Department of Dermatology The Johns Hopkins Hospital Baltimore Maryland USA

**Keywords:** noninvasive imaging, optical coherence tomography, pigmentary disorders, vitiligo

To the Editor,

Pigmentary disorders, particularly vitiligo, present significant challenges in dermatological treatment. Accurate monitoring of therapeutic efficacy is essential for optimizing treatment strategies. Noninvasive imaging offers a promising solution for real‐time evaluation of skin changes, enabling clinicians to assess treatment outcomes while limiting invasive procedures. This manuscript analyzes the application of noninvasive imaging modalities, specifically optical coherence tomography (OCT), in assessing therapeutic responses in patients with vitiligo.

OCT integrates ultrasonography and optical interferometry to image skin horizontally and vertically up to 1–2 mm deep, capable of generating 3D images, particularly beneficial for deeper basal cell carcinoma subtypes and monitoring treatment responses while limiting additional biopsies, such as verifying clear margins and assessing therapeutic efficacy at the cellular level [1, 2]. OCT is emerging as a practical, noninvasive tool for evaluating skin characteristics and tracking surgical treatment responses in patients with pigmentary disorders, such as vitiligo. Vitiligo is characterized by autoimmune‐mediated loss of melanocytes and impaired melanin synthesis. OCT imaging can visualize skin changes by revealing decreased or absent scattering contrast between the stratum basale and dermal papillae. Recent studies demonstrate OCT's efficacy in assessing repigmentation post‐skin grafting, detecting signs as early as Day 30 [1].

In comparison, techniques such as reflectance confocal microscopy (RCM) quantify vitiligo treatment response at a cellular level, capturing melanocyte migration across submillimeter scales [3]. RCM visualizes cellular features in the epidermis, dermal–epidermal junction, and papillary dermis at near‐histological resolution, using differential refractive indices to capture grayscale images up to 300 μm deep horizontally. This allows for enhanced diagnostic accuracy for melanocytic and non‐melanocytic skin cancers, aiding in the diagnosis of inflammatory and infectious skin disorders [3]. The VivaScope 1500 RCM device, with its high lateral resolution (~1 μm), limited imaging depth (~200–250 μm), and mosaic field of view (~8 × 8 mm), excels in detailed visualization of epidermal and dermo‐epidermal structures, such as melanocytes and keratinocytes, making it particularly effective for assessing cellular‐level repigmentation in vitiligo [4]. In contrast, ultrahigh‐resolution OCT, with a lower lateral resolution (~5–15 μm) but greater imaging depth (~1–2 mm) and comparable field of view (~8 × 8 mm), is better suited for efficiently monitoring structural changes in dermal papillae and larger‐scale tissue alterations post‐treatment (Table 1) [1, 4].

**TABLE 1:** Comparison of RCM and OCT parameters for vitiligo treatment monitoring.

Imaging technique	Spatial resolution	Imaging depth	Field of view
Reflectance confocal microscopy (RCM)^a^	~1 μm	200–250 μm	Single frame: 500 μm × 500 μm Mosaicked field: up to 8 mm × 8 mm
Optical coherence tomography (OCT)^b^	4.5 μm	1–2 mm	8 × 8 mm

^a^ As per the imaging settings of the Lucid VivaScope 1500 used by Ardigo et al. [4]

^b^ As per the imaging settings of the high‐resolution OCT system used for vitiligo treatment monitoring by Xie et al. [1]

Case studies using OCT for real‐time monitoring of epidermal and dermal changes in vitiligo show promising results. One study investigated high‐resolution full‐field optical coherence tomography (CRFF‐OCT) for assessing vitiligo lesions post‐tissue grafting in 10 patients [2]. Over 6 months, clinical, dermoscopic, and photographic data revealed melanin loss as a distinct dark band in vitiligo lesions using CRFF‐OCT. Grafted areas exhibited melanocytes with dendrites around the epidermal–dermal junction and hair follicles. CRFF‐OCT effectively detected early melanocyte recovery and accurately quantified melanin [2]. OCT detected early melanin recovery, which validated treatment efficacy and guided clinical decisions by precisely assessing pigmentary changes, showing promise in enhancing diagnostic accuracy and optimizing therapeutic regimens in vitiligo [2].

Other promising noninvasive imaging tools for post‐treatment vitiligo monitoring include fluorescence‐advanced videodermoscopy (FAV) for visualizing pigmented cell proliferation at the dermal–epidermal junction and high‐frequency ultrasound (HFUS) for detecting subclinical inflammatory patterns [5, 6].

Noninvasive imaging techniques, particularly OCT, are of growing importance in the management of pigmentary disorders such as vitiligo by enabling precise and quantifiable monitoring of treatment responses. Future research, particularly on combining OCT with multispectral imaging or confocal microscopy may offer deeper insights into skin pigmentation changes and treatment outcomes (Table 2). Enhancing imaging resolution and depth through artificial intelligence and machine learning, as well as exploring OCT's role in targeted drug delivery, holds potential for advancing dermatological care. Continued research is essential to optimize OCT and integrate it effectively with complementary tools.

**TABLE 2:** Potential future directions in OCT research for dermatological applications.

Future research direction	Description
AI and machine learning integration	Utilize AI algorithms to enhance OCT image analysis, automate detection of skin features, and improve diagnostic accuracy
Targeted drug delivery	Investigate OCT‐guided techniques for precise delivery of therapeutic agents to specific skin layers or lesions
Assessment of new therapeutic agents	Evaluate OCT's capability to monitor the effects of dermatological treatments at a cellular level in clinical testing
Integration with multispectral imaging	Combine OCT with multispectral imaging to comprehensively characterize changes in skin pigmentation
Enhance imaging resolution and depth	Develop OCT systems with higher resolution and deeper penetration to visualize finer skin features, structures, and tissue layers

## Consent

The authors have nothing to report.

## Conflicts of Interest

The authors declare no conflicts of interest.

## Data Availability

The data that support the findings of this study are available from the corresponding author upon reasonable request.
